# Bacterial Cellulose Powder from Tropical Fruit Byproducts: Characterization and Application in Smoothies

**DOI:** 10.17113/ftb.64.02.26.8999

**Published:** 2026-06-15

**Authors:** Rafael Souza Cruz, Giovana Matias do Prado, Paulo Henrique Machado de Sousa, Nágila Maria Pontes Silva Ricardo, Débora Hellen Almeida de Brito, Francisco Ernani Alves Magalhães, Jéssica Azevedo Furtado, Larissa Morais Ribeiro da Silva

**Affiliations:** 1 Federal University of Ceará, Campus do Pici, Ac. Público, 856 - Pici, 60020-181 Fortaleza, CE, Brazil; 2 Laboratory of Bioprospection of Natural Products and Biotechnology (LBPNB), 63660-000, Rua Solon Medeiros, S/N, BR 020, Bairro Bezerra e Sousa, Tauá, Ceará, Brazil

**Keywords:** kombucha, co-product, powder, bacterial cellulose, industrial reuse

## Abstract

**Research background:**

The development of new products based on bacterial cellulose powder derived from tropical fruit byproducts (pulp and peels) represents a technological alternative that offers environmental benefits to everyone. This solution can be applied in both industrial and domestic settings. In this research, bacterial cellulose was produced by fermentation of industrial waste from tropical fruits.

**Experimental approach:**

Bacterial cellulose powders were produced *via* kombucha fermentation using agro-industrial byproducts from tropical fruits. The powders were selected and characterized according to physicochemical parameters, proximate composition, bacterial count, Fourier transform infrared spectroscopy (FTIR), thermogravimetric analysis (TGA) and *in vivo* toxicity.

**Results and conclusions:**

The powders had pH values from 2.5 to 4.5. Acerola bacterial cellulose showed the highest yield (6.25 %) and the highest vitamin C mass fraction ((1998±51) mg/100 g). Pseudoplastic behavior was observed in all smoothies, and the formulation containing bacterial cellulose from passion fruit showed the highest viscosity among the evaluated samples. Zebrafish tests did not indicate any adverse effects related to the formulations.

**Novelty and scientific contribution:**

The use of bacterial cellulose powders from agro-industrial waste could be a healthy and sustainable alternative for the development of new products with a high vitamin C content (acerola bacterial cellulose powder) or more viscous products (passion fruit bacterial cellulose powder).

## INTRODUCTION

Kombucha is widespread in both the East and West due to its popularity and association with various therapeutic benefits (*1*). It is a beverage fermented from *Camellia sinensis* tea leaves by bacteria and yeast, which produce bacterial cellulose – a cellulosic film known as the symbiotic culture of bacteria and yeasts (SCOBY). This film forms on the surface of the liquid during fermentation (*2*) and consists of a range of microorganisms from different genera, such as *Gluconobacter*, *Acetobacter*, *Zygosaccharomyces* and *Saccharomyces* (*3*).

During fermentation, yeasts consume sucrose, hydrolyzing it into glucose and fructose, which are subsequently converted into ethanol and carbon dioxide. Meanwhile, bacteria produce gluconic acid and acetic acid. A wide variety of compounds are present in this beverage, including water-soluble vitamins, amino acids, pigments, lipids, proteins, hydrolytic enzymes, ethanol, polyphenols, minerals, and metabolic products of yeast and bacteria (*1*, *4*). Many of these compounds remain embedded in the bacterial cellulose, for which alternative uses must be considered as the beverage industry grows.

In recent years, several applications for this bacterial cellulose have been investigated, demonstrating significant potential for future use (*5*). For example, kombucha bacterial cellulose has been used to create a new beer with acidic characteristics and high antioxidant activity (*6*). Additionally, nanoparticles have been produced using yeast isolated from kombucha bacterial cellulose (*7*).

The growing consumption of fermented products worldwide has encouraged the food industry to explore alternatives that diversify these products, offering consumers new food options (*8*-*10*) and consequently generating waste from these processes. Since fermentation is based on sugar consumption through the symbiotic association of bacteria and yeast, it is possible to envision kombucha being prepared with different substrates beyond traditional tea (*3*).

Smoothies are an effective way to promote fruit and vegetable consumption. These beverages are considered rich sources of bioactive compounds and offer numerous health benefits (*11*). Although smoothie consumption is already associated with various health benefits, numerous studies have focused on increasing their bioactive compound content to create products with greater functional value. For example, a strawberry and apple smoothie was developed by adding plant extracts, resulting in higher antioxidant activity (*12*). Similarly, a smoothie made from persimmon purée and apple was enhanced with plant extracts (*13*), and a strawberry smoothie with pomegranate extract was evaluated for its biological activities (*14*).

In this research, waste from the tropical fruit processing industry was used as a carbon source to produce bacterial cellulose through kombucha fermentation. Its potential as a new natural additive was evaluated to enhance viscosity and improve the nutritional value of smoothie-type beverages.

The selection of fruit processing waste was based on market availability. specifically, the pulp most consumed by the population, which consequently generate the largest amount of organic matter. This approach enables the production of other foods, thereby reducing environmental impact and organic waste (*15*, *16*).

The development of new foods, such as smoothies enriched with bacterial cellulose derived from tropical fruit waste, is a technological alternative that provides environmental benefits and can be applied in both industrial and artisanal settings. With this in mind, the selection of raw materials for fermentation was based on the most commercially relevant fruits, which generate the greatest amount of industrial waste. Furthermore, sustainable food use reduces the production of organic waste and is associated with the creation of new foods, making it a promising alternative across different areas of the food industry.

This study aims to obtain bacterial cellulose from the fermentation of tropical fruit residues. The bacterial cellulose produced was characterized and applied to a smoothie-type product.

## MATERIALS AND METHODS

### Raw material

Tropical fruit waste from acerola (*Malpighia emarginata*), araçá (*Psidium cattleianum*), pineapple (*Ananas comosus*), guava (*Psidium guajava*), mango (*Mangifera indica*) and passion fruit (*Passiflora edulis*) was provided by an industry (Nossa Fruta Brasil, Eusébio, CE, Brazil), from the 2021/2022 harvest and collected in plastic packaging shortly after pulp processing.

Green tea (Dr. Oetker®, São Paulo, SP, Brazil), sugar (União®, São Paulo, Brazil) and milk (Betânia, Fortaleza, Ceará, Brazil) used to prepare the smoothie were purchased at local market stores in Fortaleza, CE, Brazil. The samples were registered in the National System for the Management of Genetic Heritage and Associated Traditional Knowledge (SISGEN) under accession number AA72205 through the Federal University of Ceará, Fortaleza, Brazil.

The initial cellulosic film used in this study was donated by our research group in Fortaleza, CE, and it is not possible to identify the production properties of the ’mother of kombucha’. The bacterial cellulose obtained was kept refrigerated in the kombucha itself in a glass container until use. During the experiment, the bacterial cellulose was divided into portions to be placed in each container with the previously mentioned extracts of tropical fruit co-products, and each container was subjected to a new fermentation medium for 7 days before use.

### Use of fruit residues as an alternative substrate in the fermentation of kombucha

**Fig. S1:**
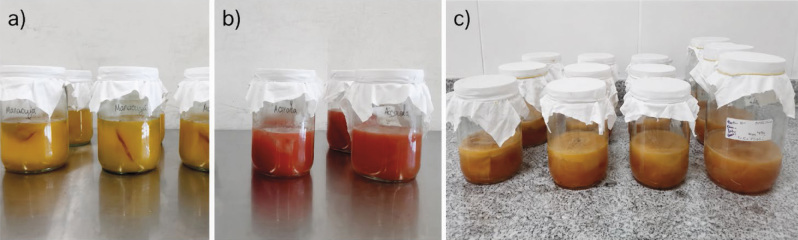
Initial fermentation process. The containers show the resting byproducts from: a) passion fruit, b) acerola, and c) green tea

Preliminary tests were carried out with different fruit byproducts (Fig. S1) to evaluate fermentation and the potential use of these byproducts for fermentation and the production of bacterial cellulose. For the fermentation process, both the symbiotic culture of bacteria and yeasts (SCOBY) and the liquid from the end of the kombucha fermentation test (prepared in advance) were used as the starter culture (provided by our research group in Fortaleza, Brazil). It is important to note that using cultures from traditional fermented products such as kombucha and kefir may produce different results.

The preparation of the formulations starts with the infusion phase (at (90±2) °C for 5 min) using drinking water, the specific fruit byproducts (13 % *m*/*V*) and 7 % sugar. After the infusion phase, the samples were filtered through felt tissue to remove solid residues. The resulting liquid was then cooled ((24±2) °C) and 10 and 20 % (*m*/*V*) kombucha bacterial cellulose (SCOBY) were added, starting the fermentation process in the presence of oxygen. The bacterial cellulose used for all formulations was from the same initial fermentation, and the same amount of this culture was used for all formulations. A kombucha was also prepared using green tea as a control. The fermentation process was carried out for 7 days at 35 °C (data not shown).

**Fig. S2:**
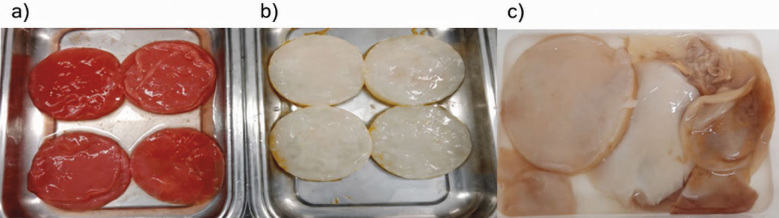
Obtaining bacterial cellulose from: a) acerola, b) passion fruit, and c) green tea substrate

After fermentation of the formulations, cellulose formation was observed (Fig. S2), followed by the dehydration stage to obtain the powder yield.

### Obtaining powders from selected bacterial celluloses

For the bacterial cellulose freeze-drying process, the methodology followed that described by Nunes *et al.* (*17*), in which the samples were frozen and freeze-dried (Liobras, São Paulo, Brazil) for 48 h at -50 °C. The freeze-dried material was crushed in a mortar using liquid nitrogen, as the freeze-drying process was inefficient due to the high sugar concentration in the cellulose. To preserve the material, low-density polyethylene packaging bags wrapped in aluminum foil were used and placed in a desiccator until analysis.

### Characterization of kombucha bacterial cellulose powders

The powders obtained were subjected to physicochemical analyses (humidity, pH, soluble solids, total titratable acidity, vitamin C content, water activity, FTIR and TGA), as well as microbiological and toxicological analyses.

### Physicochemical and yield analyses

When obtaining the powdered material, the yield was determined using the following equation, described by Andrade *et al.* (*18*), with modifications:


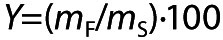
 /1/

where *Y* is the yield (%), *m*_F_ is the dry mass (g), and *m*_S_ is the mass of the byproduct before drying (g).

Humidity was determined based on the moisture loss of samples dried in an oven (SSD 30L; SolidSteel Ltda., Piracicaba, SP, Brazil) at 105 °C until constant mass (*19*).

The pH was determined potentiometrically with a digital pH meter (model 3505; Jenway, Stone, UK) calibrated with pH buffer solutions at 4.0 and 7.0 (*19*).

Soluble solids were measured using a portable digital refractometer (model RT 32; ASKO, São Leopoldo, Brazil) and the results were expressed in °Brix (*19*).

Total titratable acidity was determined by potentiometric titration with 0.1 M NaOH under stirring until pH=8.1, and the results were expressed as percentage of malic acid (*19*).

Ascorbic acid content was determined by titration with a 0.02 % 2,6-dichlorophenolindophenol (DFI) solution until a permanent light pink color, using 5 g of the sample diluted in 50 mL of oxalic acid (0.5 %), according to Strohecker and Henning (*20*). The results were expressed in mg/100 g of ascorbic acid (*19*).

Water activity (*a*_w_) was measured directly using an Aqualab instrument (Aqualab LITE; Decagon, Pullman, WA, USA), with activated carbon as a control blank (*19*).

### Fourier-transform infrared spectroscopy analysis

Fourier transform infrared spectroscopy (FTIR) analysis was conducted using an IRTracer-100 (Shimadzu, Kyoto, Japan) spectrometer in the infrared range between 4000 and 400 cm^-1^ with a resolution of 4 cm^-1^ at room temperature, on a KBr pellet. Each sample was scanned 64 times to verify similarities in the composition, following the methodology described by Fontes *et al*. (*21*). The samples (in triplicate) were divided into two groups according to the pre-established formulations based on the mass yields of bacterial cellulose.

A range of 400 to 450 cm^-1^ (mid-infrared region) was established with a resolution of 4 cm^-1^ at room temperature, and the infrared spectra were subjected to multivariate analysis to assess similarity among the different samples (*21*).

### Thermogravimetric analysis

Thermogravimetric analysis (TGA) was conducted to assess the thermal stability of the components, highlighting mass losses in different temperature ranges. TGA curves were obtained using a TGA Q50 (TA Instruments, New Castle, DE, USA) at a heating rate of 10 °C/min over a temperature range of 25 to 900 °C, with an air flow rate of 60 mL/min and an initial sample mass of approx. 10 mg.

### Microbiological analyses

Microbiological analyses were carried out using classical plate count methods with serial dilutions, with peptone water (Kasvi®, Curitiba, Brazil) as the diluent. Samples were inoculated using surface plating technique for the enumeration of total mesophilic aerobic bacteria, yeasts, and acetic acid bacteria, on plate count agar (PCA; Kasvi®), Sabouraud dextrose agar with chloramphenicol (SDA-Chl; Kasvi®), and glucose yeast extract calcium carbonate (GYC; HiMedia®, Mumbai, India) medium, incubated at 35 °C for 48 h, 22 °C for 5 days, and 30 °C for 72 h, respectively. For enumeration of lactic acid bacteria, the pour plate technique was used with de Man, Rogosa and Sharpe (MRS) agar (Kasvi®), incubated at 32 °C for 72 h. Results were expressed as colony-forming units per gram (CFU/g). Total coliforms, thermotolerant coliforms, and *Escherichia coli* counts were determined by the most probable number (MPN) method, using lactose broth (HiMedia®) incubated at 35 °C for 48 h for the presumptive test, and 2 % Brilliant Green bile (BGB; HiMedia®) broth and *E. coli* broth (EC; HiMedia®) for confirmation of total and thermotolerant coliforms, incubated at 35 °C for 48 h and 45.5 °C for 24 h, respectively. Growth with gas production was considered as confirmation of the presence of total coliforms and thermotolerant coliforms (*22*-*24*).

### Assessment of acute and locomotor toxicity of bacterial cellulose powders using zebrafish as an in vivo model

To assess toxicity, tests were carried out on zebrafish (*Danio rerio*) using the methodology proposed by Magalhães *et al.* (*25*). Adult wild zebrafish of both sexes, aged 60 to 90 days, measuring (3.5±0.5) cm and weighing (0.4±0.1) g, were used. The fish were obtained from Agroquímica: Comércio de Produtos Veterinários LTDA, a supplier in Fortaleza (Ceará, Brazil). After obtaining the *in vivo* models, groups of 50 fish were acclimatized for 24 h in glass aquaria (40 cm×20 cm×25 cm) containing dechlorinated water (ProtecPlus® antichlorine) and air pumps with submerged filters, at 25 °C and pH=7.0, with a 14:10 h light/dark circadian cycle. The fish received food (Spirulina®; NaturGreen, Madrid, Spain) *ad libitum* 24 h before the experiments.

The open-field test was carried out to evaluate changes, or the lack of them, in the motor coordination of fish, whether resulting from sedation and/or muscle relaxation (*26*). The animals (*N*=6 per group) were randomly selected, transferred to a damp sponge, and orally administered 20 µL of bacterial cellulose powder (vehicle group) (*27*). A group of untreated animals, referred to as the naïve group, was included. After treatments, the animals were placed individually in glass beakers (250 mL) containing 150 mL of aquarium water to rest. After 1 h, the animals were transferred to glass Petri dishes (10 cm×15 cm) containing the same aquarium water, each marked with four quadrants to analyze locomotor activity by counting line crossings. Using the line crossing value of the naïve group as a baseline (100 %), the percentage of locomotor activity (AL/%) was calculated individually during 5 min.

The acute toxicity study was conducted with adult zebrafish (*D. rerio*) according to the methodologies proposed by the Organization for Economic Co-operation and Development (*28*, *29*). The animals (*N*=6 per group) were treated with the same amounts of powders used in the open field test, but the fish were left to rest for 96 h to assess mortality. The vehicle group (sterile distilled water) served as the control. After 96 h, the number of dead fish in each group was recorded to determine the lethal concentration that killed 50 % of the animals (LC50), using the trimmed Spearman-Karber method with a 95 % confidence interval (*30*).

### Application of powders in the smoothie

After homogenizing the ingredients using a home blender (300 W; Arno, São Paulo, SP, Brazil), the drink was packaged in 1000-mL PVC bottles, sealed, and stored under refrigeration ((6±2) °C) until analysis.

The beverage formulations were defined through preliminary tests. The bacterial cellulose powders used were selected based on yield assessment after the fermentation period. Acerola, passion fruit, and green tea were chosen; the results of the preliminary tests that led to this selection are presented below.

**Fig. S3:**
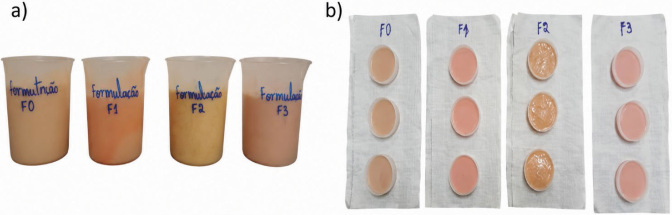
Processing of beverages with the addition of the developed bacterial cellulose powders: a) smoothie without added bacterial cellulose powder (F0), and with the addition of bacterial cellulose powder from acerola (F1), passion fruit (F2), and green tea (F3) kombucha, and b) homogenized smoothie formulations

The experiment was conducted using four smoothie formulations, resulting in a beverage without added bacterial cellulose powder (F0), and with the addition of bacterial cellulose powder from acerola (F1), passion fruit (F2), and green tea (F3) kombucha, each produced in triplicate (Fig. S3). These were later subjected to chemical and microbiological analyses. Smoothies were prepared by mixing milk (30 %), frozen fruit pulp (30 % strawberry and 20 % banana), and bacterial cellulose powder (20 %). The mixture was homogenized using a domestic blender (300 W; Arno). After homogenizing the ingredients, each beverage was packaged in 1000-mL PVC bottles, sealed, and stored under refrigeration ((6±2) °C) until analysis. The preparation of the smoothie formulations followed the basic requirements of Good Manufacturing Practices.

### Smoothie characterization

The smoothie formulations were analyzed for proximate composition. Protein mass fraction was measured by determining total nitrogen (*19*) using the Kjeldahl digestion method (Tecnal, Piracicaba, SP, Brazil). The main steps were digestion, distillation, and titration.

For lipid mass fraction, the Soxhlet method (*19*) was used, which is based on continuous extraction of lipids with the organic solvent hexane Dinâmica® (Indaiatuba, SP, Brazil) using a lipid Soxhlet extractor (Tecnal, Piracicaba, SP, Brazil), followed by the removal of the solvent by distillation, drying of the material in an oven (SolidSteel Ltda.) until constant mass, and weighing of the residual material obtained.

Ash content (*19*) was determined by weighing the material before and after heating in a muffle furnace (SolidSteel Ltda.) at 550–570 °C until constant mass was achieved.

Carbohydrate content was estimated by difference, based on the determined values of the other constituents (moisture, ash, lipids, and proteins).

The rheological behavior of the smoothies was determined using a Brookfield Searle-type rotational rheometer with concentric cylinders, model R/S plus SST 2000 (Brookfield, MA, USA). The DG-DIN sensor was used. Rheological analyses were obtained by varying the strain rate from 108 to 500 s^-1^ (ascending curve) and from 500 to 100 s^-1^ (descending curve), with a time of 1 min, and a reading of 25 points for each curve. Readings were taken in triplicate, and a new sample was used for each measurement (*31*).

### Statistical analysis

The results of the physicochemical analyses were expressed as a mean value and standard deviation, subjected to analysis of variance and Tukey's test at a 5 % level of significance using the Statistica® v. 7 (*32*) with a significance level of 5 % (p≤0.05), and Excel (*33*) was used to tabulate the obtained data.

## RESULTS AND DISCUSSION

### Preliminary tests to evaluate the production of bacterial cellulose using fruit byproducts

The substrates that showed the best performance in cellulose formation were passion fruit, acerola, and traditional green tea, while araçá, guava, pineapple, and mango substrates produced the lowest masses (data not shown). The three best substrates, based on cellulose mass produced, were selected for characterization and application in smoothies.

At the end of fermentation, the cellulose membranes exhibited different wet masses: araçá 57.00 g, pineapple 39.50 g, acerola 66.70 g, guava 41.22 g, mango 25.40 g, passion fruit 81.90 g, and green tea 60.80 g. The yeasts and bacteria inoculated into the beverage for fermentation are responsible for the growth of what is known as tea fungus, or bacterial cellulose. Acetic bacteria produce a cellulose network as a secondary fermentation metabolite, resulting in a structure that resembles a mushroom (*3*). Initially, cellulose-producing microorganisms increase in population and consume dissolved oxygen. As the microbial population increases, cellulose production also increases on the container surface. Over time, the membrane thickness increases as new layers form on its surface, creating suspended structures. In most formulations tested, an increase in fermentation time was associated with a reduction in soluble solid content (Table 1) and a decrease in pH (increase in acidity), as is common in most fermentation processes.

**Table 1:** Control parameters during the evaluated fermentation period

*t*/day	Acerola		Passion fruit		Green tea	
	TSS/°Brix	pH	TSS/ºBrix	pH	TSS/°Brix	pH
0	12.5±0.1	3.5±0.2	13.1±0.2	3.8±0.1	11.3±0.3	.5±0.1
1	12.5±0.3	3.5±0.1	12.0±0.2	3.8±0.2	30.0±0.1	4.5±0.1
2	11.01±0.05	3.0±0.2	10.9±0.2	3.2±0.4	27.53±0.05)	4.2±0.1
3	11.0±0.2	2.8±0.1	10.1±0.3	2.8±0.2	25.0±0.3	3.8±0.1
4	10.4±0.2	2.7±0.3	9.7±0.1	2.6±0.1	19.8±0.4	3.5±0.2
5	10.1±0.4	2.5±0.4	9.5±0.2	2.6±0.1	19.3±0.3	3.2±0.1
6	9.5±0.2	2.5±0.2	9.50±0.09	2.5±0.1	18.0±0.2	2.9±0.1
7	9.5±0.1	2.5±0.1	8.8±0.1	2.5±0.1	17.2±0.4	3.0±0.2

TSS=total soluble solids

For the control sample (fermented with green tea), this behavior was not observed for the soluble solid content. However, a reduction in pH was observed, indicating that fermentation occurred. The final pH values for the formulations ranged from 2.5 to 3.0.

After 24 h of fermentation, films formed on the surfaces of some containers. According to Goh *et al.* (*34*), these variations in bacterial cellulose production depend greatly on the strains used, fermentation time, and the chemical compounds present in the fermentation medium.

At the end of fermentation (7 days), cellulose production was evaluated by measuring the mass of bacterial cellulose produced. The biofilms were removed from the containers and washed with distilled water to remove impurities. After washing the membranes and removing excess water, they were dehydrated, then characterized and applied in the smoothie.

Acerola bacterial cellulose powder showed the highest yield (6.25 %), followed by passion fruit bacterial cellulose powder (3.84 %) and green tea bacterial cellulose powder (3.95 %). The acerola bacterial cellulose yield demonstrated greater productivity in terms of mass. This may be related to the composition of the acerola residue, which is rich in dietary fiber, including cellulose, hemicellulose, and pectin. This characteristic may have contributed to better bacterial cellulose yields, as all samples were subjected to the same incubation conditions for fermentation.

### Influence of different fruit byproducts on the physicochemical properties of bacterial cellulose powder

The acidity values of bacterial cellulose powders after fermentation ranged from 3.2 mmol/L for the bacterial cellulose powder with acerola and 3.8 mmol/L for the passion fruit bacterial cellulose powder to 4.2 mmol/L for the green tea bacterial cellulose powder, as shown in Table 2. Food pH is considered an indicator of food safety. A pH within the acidic range, below 4.5, inhibits the growth of the main microorganisms responsible for foodborne diseases (*35*).

**Table 2:** Physicochemical characterization of kombucha bacterial cellulose powders

Parameter	Acerola bacterial cellulose	Passion fruit bacterial cellulose	Green tea bacterial cellulose
TTA/(mmol/L)	(3.2±0.3)^a^	(38±0.4)^a^	(4.2±0.2)^a^
pH	(2.77±0.04)^a^	(2.9±0.1)^a^	(2.90±0.00)^a^
*w*(vitamin C)/(mg/100 g)	(1998±51)^a^	(163.3±3.1)^c^	(393±4)^b^
TSS/^o^Brix	(6.7±0.3)^a^	(6.13±0.06)^ab^	(8.13±0.06)^c^
*w*(protein)/%	(1.4±0.2)^a^	(1.4±0.2)^a^	(0.6±0.3)^a^
*w*(ash)/%	(2.6±3.3)^a^	(1.1±0.5)^a^	(2.2±0.2)^a^
*w*(lipid)/%	(0.56±0.00)^a^	(0.23±0.00)^a^	(0.6±0.0)^a^
*a*_w_	(0.22±0.00)^a^	(0.28±0.01)^a^	(0.24±0.01)^a^

TTA=total titratable acidity, TSS=total soluble solids

When comparing the results obtained from the powders with the normative instruction for kombucha (*36*), it is evident that the acidity and pH analyses are consistent with the findings of this research: acidity ranged from 3.2 to 4.2 mmol/L(p>0.05), and pH ranged from 2.8 to 2.9, for the acerola, passion fruit, and green tea bacterial cellulose powders.

Regarding vitamin C content, the highest value was observed in the acerola bacterial cellulose powder ((1998±51) mg/100 g), followed by the green tea bacterial cellulose powder ((393±4) mg/100 g), and finally the passion fruit bacterial cellulose powder ((163.3±3.1) mg/100 g). Vitamin C is an essential nutrient that plays an important role in the human body. Its presence in kombucha, especially when derived from acerola residue, can provide additional benefits to the beverage and contribute to its classification as a functional beverage, while also helping to preserve the product through its antioxidant properties. Furthermore, the presence of vitamin C may be a microbial growth factor to consider for bacterial cellulose mass yield. The consumption of foods rich in vitamin C has been increasing, as it is directly associated with delaying cellular aging and reducing the incidence of degenerative diseases, cardiovascular diseases, inflammation, brain dysfunction, and other conditions. The recommended value for vitamin C is 45 mg/day (*37*). It is noteworthy that the vitamin C content in the acerola bacterial cellulose powder was over five times greater than that in bacterial cellulose powder from green tea, demonstrating its potential use in the development of new foods with a higher content of this vitamin.

Soluble solids were measured at (6.7±0.3) °Brix for acerola bacterial cellulose powder, (6.13±0.06) °Brix for passion fruit bacterial cellulose powder and (8.13±0.06) °Brix for green tea bacterial cellulose powder. During fermentation, soluble solids may decrease as sugars are consumed by bacteria and yeast in the bacterial cellulose. This measurement is useful for controlling the sugar and nutrient content of the final beverage (*38*).

Studies show that new raw materials can serve as alternative substrates for the fermentation of beverages similar to kombucha. In the research conducted by Câmara *et al.* (*39*), residues of red guava, pineapple, cashew, mango, and mombim were used as alternative substrates for kombucha production. Analysis of the proximate composition highlighted the high nutritional value of these raw materials, which influenced the preparation of kombuchas.

The protein mass fractions of the green tea, passion fruit, and acerola bacterial cellulose powder samples were below 2 %, with the latter two showing higher protein mass fractions (around 1.4 %) than the green tea (0.6 %). Research by Moraes *et al.* (*40*) reported a protein value of 0.20 g/100 mL in the passion fruit kombucha sample, which is lower than the result obtained in this study.

According to the Brazilian Food Composition Table (*41*), 100 g of passion fruit contains 0.8 g of protein, a value that is added to its bacterial cellulose powder, and 100 g of acerola contains 0.4 g of protein.

The samples showed low lipid mass fractions (*w*=0.23–0.60 %), which may be related to the low lipid content of fruits and green tea used (*41*). Bacterial cellulose powders produced with the addition of fruit co-products would contribute only around 0.66 % to lipid intake.

The relative ash values obtained in this study were from 1.1 to 2.6 %. These values are lower than those reported by Silva *et al.* (*42*), who found values between 4.59 and 7 %.

Water activity (*a*_w_) is an important factor in evaluating food stability, as it corresponds to the thermodynamically available water for chemical and biochemical reactions (*43*). Furthermore, *a*_w_ can provide important data on the moisture content of raw materials.

**Fig. 1:**
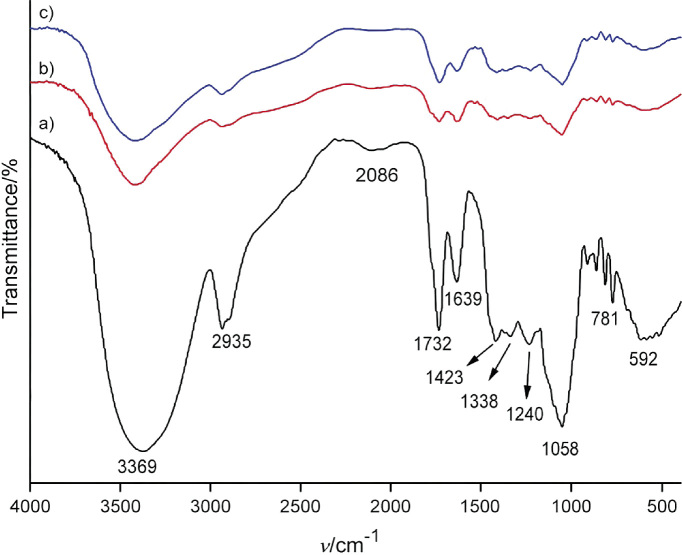
FTIR spectra of acerola (a), passion fruit (b) and green tea (c) bacterial cellulose powder

The FTIR spectra (Fig. 1) showed similarities among the three samples and were consistent with the chemical structure identified in bacterial cellulose derived from acerola byproduct, as reported by Leonarski *et al.* (*44*). The analysis revealed characteristic bands associated with bacterial cellulose, including an OH-band at approx. 3369 cm^−1^ and a CH stretch of CH_2_ and CH_3_ groups around 2935 cm^−1^. Bands near 1639 and 1423 cm^−1^ were attributed to the glucose carbonyl group (C=O), as well as CH_2_ bending and C–OH in-plane bending. Additionally, the spectral region between 1338 and 1240 cm^−1^ indicated the presence of crystalline regions within the cellulose structure.

Carbohydrates, such as cellulose and other polysaccharides, have characteristic bands around 1000-1200 cm^-1^ (C-O-C bond region) and 3000-3600 cm^-1^ (O-H bond region). Proteins present in bacterial cellulose can exhibit characteristic bands around 1650-1700 cm^-1^ (C=O peptide bond region) and 3100-3500 cm^-1^ (N-H and O-H vibration region). Lipids can present bands in different areas, depending on their specific composition. Generally, absorption bands are observed around 2800-3000 cm^-1^ (C-H bond region) and 1700-1750 cm^-1^ (C=O bond region of fatty acids). Polyphenols, such as phenolic acids and flavonoids, can exhibit characteristic bands around 1600-1700 cm^-1^ (aromatic C=C bond region) and 3200-3600 cm^-1^ (O-H vibration region).

It is worth noting that the exact wavelengths of the bands may vary depending on the specific composition of the bacterial cellulose. In the case of passion fruit and green tea bacterial cellulose powders, the presence of functional compounds may vary depending on the unique chemical composition of the passion fruit and its interactions with the bacterial cellulose.

These are general differences in the functional compounds found in different bacterial cellulose powders. The specific composition may vary depending on the preparation method, the variety of fruit or tea used, and other factors.

### Results of thermogravimetric analysis

**Fig. 2:**
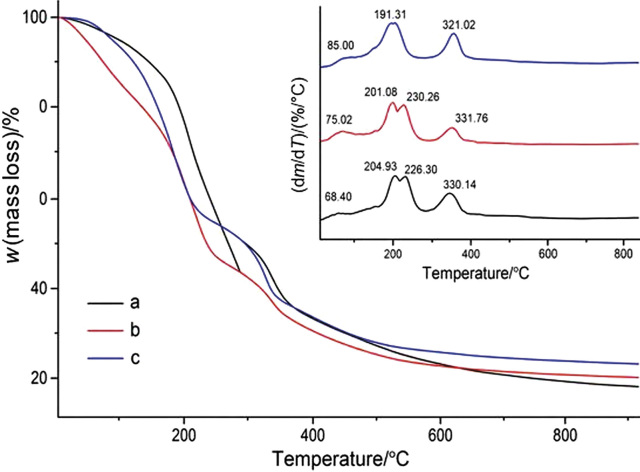
Thermogravimetric analysis of acerola (a), passion fruit (b), and green tea (c) bacterial cellulose powders

The thermogravimetric analysis (TGA) curves of acerola (a), passion fruit (b) and green tea (c) bacterial cellulose are shown in Fig. 2. All samples showed similar decomposition behavior.

The first event corresponds to sample dehydration, followed by the degradation of low-molecular-mass components, probably originating from the fermentation process. The event with initial degradation temperature (*T*_onset_) at 254 °C (a), 219 °C (b) and 245 °C (c) showed maximum degradation rate temperatures (*T*_max_) at 339 °C (a), 331 °C (b) and 321 °C (c), corresponding to the depolymerization and decomposition of glycosyl units. The total mass loss during the analysis was 81 % (a), 79 % (b) and 77 % (c). The same behavior was previously reported by Dima *et al.* (*45*).

### Influence of different fruit byproducts on the microbiological properties of bacterial cellulose powder

The results of the microbiological analyses (data not shown) indicate that the developed powders are safe for consumption from a microbial perspective, as they showed *E. coli* counts of <3 MPN/mL.

For the lactic acid bacteria count, the results were 8.0·10^2^ CFU/g for sample a, 3.0·10^2^ CFU/g for sample b, and 4.0·10^2^ CFU/g for sample c, similar to those reported by Binda and Ouwehand (*46*).

For the count of acetic acid bacteria, results of <10 CFU/g were obtained for samples a, b and c. These bacteria have been associated with several health benefits (*47*). They also play a crucial role in kombucha production, as they produce acetic acid, which gives kombucha its characteristic flavor and contributes to its preservation.

The yeast count was <10 CFU/g for samples a, b, and c. It is important to note that different yeasts may be present in bacterial cellulose, contributing to the flavor, aroma, acidity, antioxidant potential, and sensory properties of kombucha. Five yeasts were found in kombucha produced from telang flower (*Clitoria ternatea* L.) tea (*48*). No data were found in the literature on the enumeration of these microorganisms in powdered bacterial cellulose.

### In vivo toxicity of bacterial cellulose powders

**Fig. 3:**
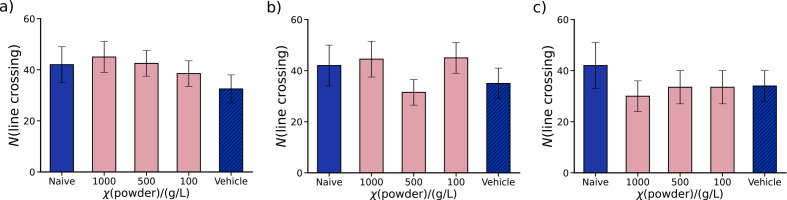
Effect of the bacterial cellulose powder of: a) acerola, b) passion fruit, and c) green tea on the locomotor activity of adult zebrafish (*Danio rerio*) in the open field test. Crossing of lines refers to samples a), b) and c) at concentrations 1000, 500 and 100 g/L. Naïve=untreated animals, p.o.=oral administration, vehicle=sterile distilled water (*V*=20 µL; p.o.). Values ​​represent the mean±standard deviation, *N*(animal)=6 (ANOVA followed by Tukey’s test)

The results of the open field test for bacterial cellulose powders from acerola (a), passion fruit (b) and green tea (c) are shown in Fig. 3. Oral administration of the powders diluted in water to animals did not cause motor impairment. This suggests that, under the experimental conditions used, ingestion of kombucha bacterial cellulose powders did not affect the animals' locomotor activity. Although there was a reduction in line crossings in the Petri dish among animals treated with bacterial cellulose powders compared to the control and naïve groups, this difference was not statistically significant.

In the acute toxicity test, no mortality was observed for acerola, passion fruit or green tea bacterial cellulose powders during the analyzed period (data not shown). Therefore, powders developed with alternative substrates (acerola and passion fruit) appear to be safe for human consumption, without exhibiting toxicity that could compromise human health.

From the results obtained, the concentrations tested for the three samples are safe, with LC50 values (lethal concentration for 50 % of organisms) above 1 mg/mL. This indicates that the samples did not cause significant mortality in the organisms tested during the 96-hour period.

An LC50 above 1 mg/mL indicates that the concentration is not lethal to at least half of the exposed organisms. This is a positive indication of safety regarding the acute effects of the samples on the organisms analyzed. Studies evaluating the toxicity of food products in adult zebrafish have been conducted, demonstrating the safety of these products for consumption (*49*-*51*).

### Influence of bacterial cellulose application in smoothies

The inclusion of bacterial cellulose from kombucha powder directly affected the vitamin C content of this product, especially with acerola bacterial cellulose powder (Table 3).

**Table 3:** Physicochemical, proximate, and vitamin C analyses of smoothie beverages with and without the addition of bacterial cellulose powder

Parameter	Smoothie			
	F0	F1	F2	F3
pH	(4.5±0.6)^a^	(4.2±0.1)^a^	(4.2±0.2)^a^	(4.3±0.3)^a^
TTA/(mmol/L)	(2.98±0.02)^a^	(3.9±0.1)^a^	(4.50±0.04)^ab^	(5.1±1.0)^bc^
TSS/°Brix	(26.7±0.1)^a^	(17.0±0.2^b^	(18.00±0.03)^b^	(16.0±0.2)^b^
*a*_w_	(0.983±0.001)^a^	(0.98±0.00)^a^	(0.981±0.001)^a^	(0.981±0.002)^a^
*w*(vitamin C)/(mg/100 g)	(51.2±0.2)^a^	(275.1±0.1)^b^	(145.63±0.04)^c^	(74.00±0.01)^ad^
*w*(humidity)/%	(11.1±0.2)^a^	(6.4±0.2)^b^	(7.35±0.02)^bc^	(7.9±0.3)^bc^
*w*(protein)/%	(2.3±0.4)^a^	(4.9±0.4)^b^	(3.6±0.1)^c^	(2.6±0.3)^cd^
*w*(ash)/%	(0.7±0.6)^a^	(3.0±0.2)^b^	(1.78±0.02)^c^	(2.6±0.6)^cd^
*w*(lipid)/%	(0.2±0.3)^a^	(0.58±0.06)^a^	(0.4±0.2)^a^	(0.4±0.1)^a^

TTA=total titratable acidity, TSS=total soluble solids, F0=control smoothie, without added bacterial cellulose powder and smoothie with F1=acerola, F2=passion fruit and F3=green tea bacterial cellulose powder. Mean values followed by a capital letter in the column and a lowercase letter in the row do not differ significantly from each other (p>0.05) according to Tukey’s test

The pH values serve as a safety parameter for food products. No significant difference in pH was found among the smoothies. Therefore, the addition of acerola, passion fruit or green tea bacterial cellulose powder did not influence this property. Regarding acidity, the formulations containing passion fruit and acerola bacterial cellulose were more acidic, which can be attributed to the inherent acidity of the powders (Table 3). The combination of fermentation, bacterial cellulose composition, time, and additional ingredients may contribute to the observed differences in smoothie acidity. In an analysis of 36 samples of smoothies made with different fruit pulps, acidity values ranged from 0.494 to 1.60 g of lactic acid per 100 g of sample (*52*).

A difference was also observed in the soluble solids content of the beverage developed without bacterial cellulose powder, which was 26.7 °Brix. In contrast, the formulations containing kombucha bacterial cellulose powder presented values of 16 to 18 °Brix, probably due to the composition of the bacterial cellulose itself, which may have influenced the arrangement of the soluble molecules present in it.

In the research carried out by Gallina *et al.* (*52*), the soluble solids values for acerola, passion fruit, strawberry, and mango smoothies ranged from 12.1 to 18.7 °Brix, similar to those found in this research.

The *a*_w_ values for all formulations were 0.98 and did not differ statistically significantly. Vitamin C mass fraction was the highest in formulations F1 and F2, which were significantly different from the other formulations at p˂0.05. This difference in vitamin C mass fractions is due to ingredients with the highest vitamin C content, such as acerola kombucha bacterial cellulose powder (1998 mg/100 g) and passion fruit bacterial cellulose powder (163.3 mg/100 g). Therefore, the inclusion of these powders, compared with the control smoothie, increases the vitamin C mass fraction.

Only the F0 formulation showed a significantly higher moisture value (11.11 %) than the other formulations. A significant difference (p<0.05) was observed in the protein mass fraction between the smoothie without added bacterial cellulose powder ((2.3±0.4) %) and the others. The smoothie with added acerola bacterial cellulose powder had a protein value of (4.9±0.4) %, standing out from the other formulations, followed by the smoothie with added passion fruit bacterial cellulose powder and the smoothie with added green tea bacterial cellulose powder. Kombucha bacterial cellulose powder is mainly composed of a cellulose matrix produced by the bacteria and yeast present in it. This matrix contains proteins that contribute to their increased mass fractions in smoothie formulations. A maximum protein mass fraction of 2.76 % was found in smoothies made with pineapple, watermelon, and mango (*53*).

Regarding the ash content of the smoothie beverages, there was a significant difference (p<0.05) between the smoothie without added bacterial cellulose powder and the smoothies with added bacterial cellulose powder. Acerola bacterial cellulose powder had a higher amount of minerals and inorganic compounds than passion fruit kombucha and green tea bacterial cellulose powders. Thus, by adding acerola bacterial cellulose powder to the smoothie, it is possible to increase the ash mass fraction (an indicator of mineral content) in the final beverage.

The lipid mass fraction in the smoothie beverage formulations did not differ significantly (p>0.05), indicating that the addition of bacterial cellulose powder did not affect this component. Although most bacterial cellulose consists of cellulose and other carbohydrates, it is also possible to find lipids in smaller quantities. Therefore, this characteristic observed in the smoothies is relevant and can be used as a nutritional appeal, making them more attractive to people interested in consuming healthier foods with low lipid and caloric content.

The smoothies were processed following good handling practices, with total and thermotolerant coliform counts below 3 MPN/g for all formulations (data not shown). For mesophilic counts, values of 10^2^ CFU/g were obtained. As the pulp used was not pasteurized and had a microbial load, it is reasonable to assume this count originates from the raw materials used to prepare the food, which is not considered high.

Treatment effects on the rheological properties of foods must be known for better process control (*54*). Understanding flow behavior is necessary to determine food viscosity. When developing smoothies, one parameter to analyze is food viscosity. Some authors include vegetables in their formulations that positively influence this parameter, such as pumpkin and carrots in a banana smoothie (*55*), or use a fruit mixture such as banana and melon smoothie (*56*).

In the formulations developed in this research, a fruit mixture already established in gastronomy (strawberry and banana) was used, and the investigation focused on whether bacterial cellulose powders could also influence the final viscosity of the product.

**Fig. 4:**
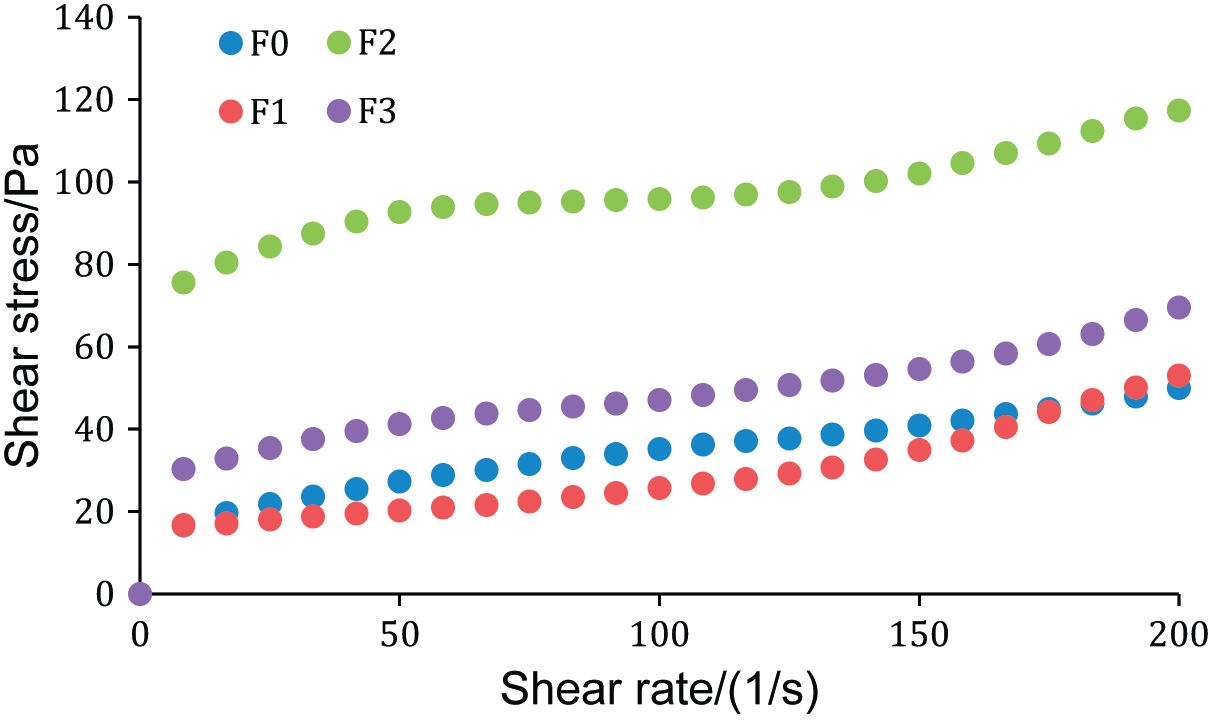
Smoothie rheograms of formulations F0 (control), F1 (acerola bacterial cellulose), F2 (passion fruit bacterial cellulose), and F3 (green tea bacterial cellulose). Viscosity as a function of shear rate

During rheological analysis, the rheometer applies a known shear force to the smoothie and measures the response of the fluid, including the shear rate and the resulting shear stress (Fig. 4). Rheological analysis of smoothies provides insights into the flow behavior of this product. It is used to adjust smoothie texture and consistency, optimize processing parameters, develop new products, or ensure the quality and consistency of the final product. Additionally, rheological analysis can help understand how ingredients, such as bacterial cellulose powder, affect smoothie properties and how they interact with other components.

The smoothie with acerola bacterial cellulose powder (Fig. 4) showed the most similar rheological behavior to the control (F0), followed by the smoothie with green tea bacterial cellulose. The smoothie containing passion fruit bacterial cellulose (F2) showed a very different rheological behavior from the other formulations, suggesting that this sample has higher viscosity than the others. It is noteworthy that the acerola residue may contain substances, such as pectin, which may have influenced the obtained result.

There are different types of non-Newtonian behavior, such as pseudoplastic, dilatant and thixotropic, among others. The flow curve for a non-Newtonian fluid may have a descending linear shape (pseudoplastic), an ascending linear shape (dilatant), or a curve with a conical shape, for example. The smoothies developed in this research showed pseudoplastic behavior.

**Fig. 5:**
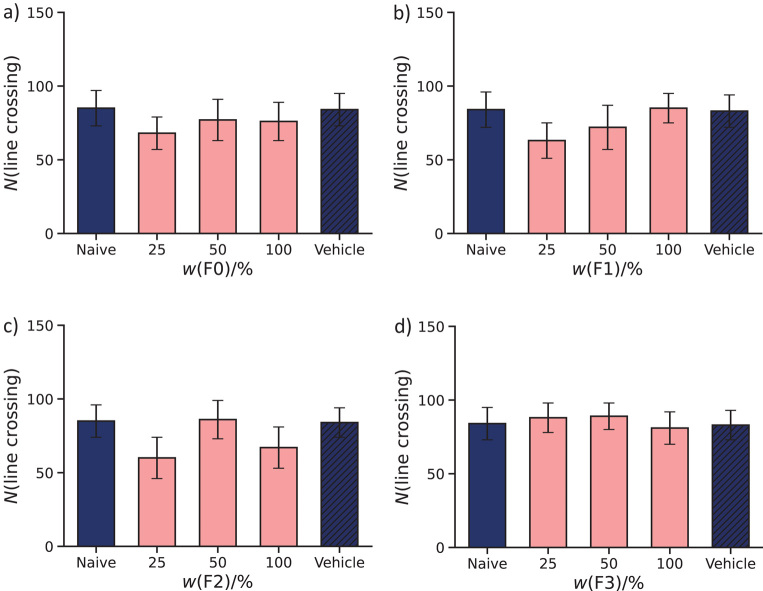
Effect of smoothie formulations (F0, F1, F2 and F3) on the locomotor activity of adult zebrafish (*Danio rerio*) in the open field test. Crossing of lines referring to the smoothie sample: a) without the addition of bacterial cellulose powder and with the addition of: b) acerola, c) passion fruit and d) green tea bacterial cellulose powder at mass fractions of 25, 50, and 100 %. Naïve=untreated animals, p.o.=oral administration, vehicle=sterile distilled water (*V*=20 µL; p.o.). Values represent the mean±standard deviation, *N*(animal)=6 (ANOVA followed by Tukey’s test)

Regarding the toxicity analysis of the smoothies (Fig. 5), there was no toxic effect in response to sample administration, a result similar to that obtained for the isolated analysis of the powders. This suggests that the formulations did not cause immediate or obvious adverse effects in fish during the open-field test.

In all samples, no mortality was recorded after 96 h, with LC50>0.25 mg/mL. Therefore, the formulations appear to be safe for human consumption, with no toxicity that could compromise human health. Although several studies have evaluated the possible sedative effects of different foods on zebrafish, more studies are needed to investigate the sedative effects and mechanisms of action of smoothies in zebrafish, and this study is one of the pioneers in the field.

No study in the literature has used this methodology to evaluate the toxicity of smoothies containing bacterial cellulose powder. Bacterial cellulose powders produced with acerola, passion fruit, and green tea byproducts have adequate physicochemical characteristics, significant mass fractions of vitamin C, relevant proximate composition, and are microbiologically safe, with acerola bacterial cellulose powder standing out. These results offer significant opportunities for the food industry, providing consumers with nutritional and sustainable options, as well as for their application in the development of smoothies. It is worth noting that the product developed is relevant to an increasingly health-conscious consumer audience, mainly used for health-related issues, older adults, or individuals with metabolic problems.

## CONCLUSIONS

The bacterial cellulose powders derived from acerola, passion fruit, and green tea showed promise, exhibiting suitable physicochemical properties, significant vitamin C content and microbiological safety. When added to smoothies, these powders significantly enriched the nutritional value of the products. Furthermore, FTIR and TG analyses provided valuable insights into the chemical changes in the smoothies resulting from the addition of bacterial cellulose, thereby improving our understanding of their composition and quality. Tests on zebrafish showed no adverse effects from the formulations, while toxicity tests confirmed the safety of the smoothies for human consumption, both pure and diluted. These results present exciting opportunities in the food industry, offering consumers nutritional and sustainable options.

## SUPPLEMENTARY MATERIAL

Supplementary material is available at https://www.ftb.com.hr/images/pdfarticles/2026/April-June/FTB-64-149-S1.pdf.

## References

[1] Miranda JF Ruiz LF Silva CB Uekane TM Silva KA Gonzalez AGM Kombucha: A review on substrates, regulations, composition and biological properties. J Food Sci. 2022;87(2):503–27. 10.1111/1750-3841.16029 35029317

[2] Abaci N Deniz FSS Orhan IE. Kombucha – An ancient fermented beverage with desired bioactivities: A narrowed review. Food Chem X. 2022;14:100302. 10.1016/j.fochx.2022.100302 35434600 PMC9011011

[3] Jayabalan R Malbaša RV Lončar ES Vitas JS Sathishkumar M. A review on kombucha tea—Microbiology, composition, fermentation, beneficial effects, toxicity, and tea fungus. Compr Rev Food Sci Food Saf. 2014;13(4):538–50. 10.1111/1541-4337.12073 33412713

[4] Leal JM Suárez LV Jayabalan R Oros JH Escalante-Aburto A. A review on health benefits of kombucha nutritional compounds and metabolites. CYTA J Food. 2018;16(1):390–9. 10.1080/19476337.2017.1410499

[5] Coelho RMD de Almeida AL do Amaral RQG da Mota RN de Sousa PHM. Kombucha [Review]. Int J Gastron Food Sci. 2020;22:100272. 10.1016/j.ijgfs.2020.100272

[6] da Silva MM de Souza AC Faria ER Molina G Andrade Neves N Morais HA Use of kombucha SCOBY and commercial yeast as inoculum for the elaboration of a novel beer. Fermentation (Basel). 2022;8(12):748. 10.3390/fermentation8120748

[7] Tsilo PH Basson AK Ntombela ZG Dlamini NG Pullabhotla RVSR. Biosynthesis and characterization of copper nanoparticles using a bioflocculant produced by a yeast *Pichia kudriavzevii* isolated from kombucha tea SCOBY. Appl Nano. 2023;4(3):226–39. 10.3390/applnano4030013

[8] Filho AALA de Sousa PHM Vieira IGP Fernandes VB Cunha FET Magalhaes FEA Kombucha and kefir fermentation dynamics on cashew nut beverage (*Anacardium occidentale* L.). Int J Gastron Food Sci. 2023;33:100778. 10.1016/j.ijgfs.2023.100778

[9] Freitas A Sousa P Wurlitzer N. Alternative raw materials in kombucha production. Int J Gastron Food Sci. 2022;30:100594. 10.1016/j.ijgfs.2022.100594

[10] Miglioranza MV Lodi KZ Minello L Aver I Magrini FE Paesi S Innovative applications based on agro-industrial residues of pitahaya for improving antioxidant and biological performance in kombuchas. Food Biosci. 2024;61:104780. 10.1016/j.fbio.2024.104780

[11] Waszkiewicz M Sokół-Łętowska A Pałczyńska A Kucharska AZ. Fruit smoothies enriched in a honeysuckle berry extract – An innovative product with health-promoting properties. Foods. 2023;12(19):3667. 10.3390/foods12193667 37835320 PMC10572983

[12] Gil KA Nowicka P Wojdyło A Serreli G Deiana M Tuberoso CIG. Antioxidant activity and inhibition of digestive enzymes of new strawberry tree fruit/apple smoothies. Antioxidants. 2023;12(4):805. 10.3390/antiox12040805 37107180 PMC10135069

[13] Gil KA Nowicka P Wojdyło A Tuberoso CIG. Investigation into polyphenol profile and biological activities of enriched persimmon/apple smoothies during storage. Foods. 2023;12(17):3248. 10.3390/foods12173248 37685183 PMC10486386

[14] Alsubhi NH Al-Quwaie DA Alrefaei GI Alharbi M Binothman N Aljadani M Pomegranate pomace extract with antioxidant, anticancer, antimicrobial, and antiviral activity enhances the quality of strawberry-yogurt smoothie. Bioengineering. 2022;9(12):735. 10.3390/bioengineering9120735 36550941 PMC9774345

[15] da Silva MBL Ramos AM. Composição química, textura e aceitação sensorial de doces em massa elaborados com polpa de banana e banana integral (Chemical composition, texture and sensory acceptance of pulp banana marmalade and whole banana marmalade). Rev Ceres. 2009;56(5):551–4. (in Portuguese)

[16] Nirmal NP Khanashyam AC Mundanat AS Shah K Babu KS Thorakkattu P Valorization of fruit waste for bioactive compounds and their applications in the food industry. Foods. 2023;12(3):556. 10.3390/foods12030556 36766085 PMC9914274

[17] Nunes MA Costa ASG Barreira JCN Vinha AF Alves RC Rocha A How functional foods endure throughout the shelf storage? Effects of packaging materials and formulation on the quality parameters and bioactivity of smoothies. LWT – Food Sci Technol. 2016;65:70–8. 10.1016/j.lwt.2015.07.061

[18] Andrade BA Perius DB de Mattos NV de Mello Luvielmo M Mellado MS. Produção de farinha de banana verde (Musa spp.) para aplicação em pão de trigo integral (Production of unripe banana flour (*Musa* spp.) for application in whole wheat bread). Braz J Food Technol. 2018;21:e2016055 (in Portuguese). 10.1590/1981-6723.5516

[19] Métodos físico-químicos para análises de alimentos (Physicochemical methods for food analysis). São Paulo, Brazil: Instituto Adolfo Lutz (IAL); 2008 (in Portuguese).

[20] Strohecker R, Henning HM. Análises de vitaminas: Métodos comprovados (Vitamin analysis: Proven methods). Madrid, Spain: Paz Montalvo; 1967 (in Spanish).

[21] Fontes V Pereira DC Pupin B Sakane KK. Aplicação de espectroscopia no infravermelho: Como ferramenta para análise quantitativa de orégano (Application of infrared spectroscopy: As tool for quantitative analysis of oregano). Rev Univap. 2020;26(51);15–25 (in Portuguese). 10.18066/revistaunivap.v26i51.2451

[22] Eaton AD, Franson MAH, editors. Standard Methods for the Examination of Water and Wastewater. Washington, DC, USA: American Public Health Association (APHA), American Water Works Association (AWWA) and Water Environment Federation (WEF); 2005.

[23] Nero LA Beloti V Barros MAF Ortolani MBT Tamanini R Franco BDGM. Comparison of Petrifilm aerobic count plates and de Man-Rogosa-Sharpe agar for enumeration of lactic acid bacteria. J Rapid Methods Autom Microbiol. 2006;14(3):249–57. 10.1111/j.1745-4581.2006.00050.x

[24] Kim DH Chon JW Kim H Seo KH. Development of a novel selective medium for the isolation and enumeration of acetic acid bacteria from various foods. Food Control. 2019;106:106717. 10.1016/j.foodcont.2019.106717

[25] Magalhães FEA Sousa CÁPB Santos SAAR Menezes RB Batista FLA Abreu AO Adult zebrafish (*Danio rerio*): An alternative behavioral model of formalin-induced nociception. Zebrafish. 2017;14(5):422–9. 10.1089/zeb.2017.1436 28704145

[26] Ahmad F Richardson MK. Exploratory behaviour in the open field test adapted for larval zebrafish: Impact of environmental complexity. Behav Processes. 2013;92:88–98. 10.1016/j.beproc.2012.10.014 23123970

[27] Collymore C Rasmussen S Tolwani RJ. Gavaging adult zebrafish. J Vis Exp. 2013;78:e50691. 10.3791/50691 23962977 PMC3855001

[28] Organization for Economic Co-operation and Development (OECD). Guideline for the testing of chemicals: Fish, acute toxicity test. Paris, France: OECD; 1992. Available from: http://www.oecd.org/chemicalsafety/risk-assessment/1948241.pdf.

[29] Huang Y Zhang J Han X Huang T. The use of zebrafish (*Danio rerio*) behavioral responses in identifying sublethal exposures to deltamethrin. Int J Environ Res Public Health. 2014;11(4):3650–60. 10.3390/ijerph110403650 24699028 PMC4024987

[30] Arellano-Aguilar O Solís-Ángeles S Serrano-García L Morales-Sierra E Méndez-Serrano A Montero-Montoya R. Use of the Zebrafish embryos toxicity test for risk assessment purpose: Case study. J Fishscicom. 2015;9(4):52–62.

[31] Brookfield Engineering Laboratories. R/S Plus Rheometer – Operating Instructions. Middleboro, MA, USA: Brookfield Engineering; 2004. Available from: https://www.brookfieldengineering.com.

[32] STATISTICA, v. 7.0, StatSoft Inc, Tulsa, OK, USA; 2004. Available from: https://www.statsoft.com.

[33] Microsoft Excel. v. 2015, Microsoft Corp., Redmond, WA, USA; 2015. Available from: https://www.microsoft.com.

[34] Goh WN Rosma A Kaur B Fazilah A Karim AA Bhat R. Microstructure and physical properties of microbial cellulose produced during fermentation of black tea broth (kombucha). Int Food Res J. 2012;19(1):153–8.

[35] Silva CFG Santos FL Santana LRR Silva MVL Conceição TA. Development and characterization of a soymilk kefir-based functional beverage. Food Sci Technol. 2018;38(3):543–50. 10.1590/1678-457x.10617

[36] Ministério da Agricultura. Pecuária e Abastecimento (MAPA). Instrução Normativa a MAPA n. 41, de 17 de setembro de 2019. Estabelece o Padrão de Identidade e Qualidade da kombucha em todo o território nacional (Normative Instruction MAPA No. 41, of September 17, 2019: Standards for identity and quality of kombucha). Brasilia, Brazil: Official Gazette of the Union Ed. 181, Section 1, p. 13; 2019.

[37] Resolução - RE nº 01, de 29 de julho de 2005. Guia para a realização de estudos de estabilidade (Resolution – RE No. 1, of July 29, 2005. Guidelines for conducting stability studies). Brasília, Brazil: National Health Surveillance Agency (ANVISA); 2005 (in Portuguese). Available from: https://bvsms.saude.gov.br/bvs/saudelegis/anvisa/2005/res0001_29_07_2005.html.

[38] Souza LFS Domingos LF Farias VL Luzia DMM. Avaliação físico-química e estabilidade do ácido ascórbico em sucos de frutas comercializados no município de Frutal, Minas Gerais (Evaluation physical-chemical and stability of ascorbic acid in fruit juices marketed in city of Frutal, Minas Gerais, Brazil). Rev Verde Agroecologia Desenvolv Sustent. 2017;12(4):791–7 (in Portuguese). 10.18378/rvads.v12i4.4184

[39] Câmara GB Prado GM Sousa PHM Lima ARN Souza Oliveira L Furtado JA Potential applicability of fruit co-products in the development of kombucha fermented beverages: A review study. Res Soc Dev. 2022;11(5):e33811525846. 10.33448/rsd-v11i5.25846

[40] Moraes LS Bender S Kottwitz LBM. Determinação composicional de amostras de kombuchas acrescidas de polpas de frutas (Compositional determination of kombucha samples added with fruit pulp). Fag J Health. 2020;2(2):252–8. (in Portuguese) 10.35984/fjh.v2i2.213

[41] Tabela brasileira de composição de alimentos - TACO (Brazilian table of food composition). Campinas, SP, Brazil: NEPA–UNICAMP; 2011. Available from: https://www.gov.br/agricultura/pt-br/assuntos/inspecao/produtos-vegetal/legislacao-programas-nacionais-e-seguranca-dos-alimentos-1/legislacao/legislacao-vinhos-e-bebidas/tabela-brasileira-de-composicao-de-alimentos_taco_2011.pdf.

[42] Silva BC Silva F Michelin DC. Avaliação da qualidade de amostras de *Camellia sinensis* (L.) Kuntze (Theaceae) comercializadas no município de Araras – SP (Assessment of quality *Camellia sinensis* (L) Kuntze (Theaceae) marketed in Araras city (SP, Brazil)). Rev Cienc Farm Basica Apl. 2013;34(2):245–50. [in Portuguese]

[43] Azeredo HMC, Brito ES, Garruti DS. Alterações químicas em alimentos durante a estocagem (Chemical changes in food during storage). In: Azeredo HMC, editor. Fundamentos de estabilidade de alimentos (Fundamentals of food stability). Brasília, DF, Brazil: Embrapa; 2012. pp. 39-77 (in Portuguese).

[44] Leonarski E Cesca K Zanella E Stambuk BU Oliveira D Poletto P. Production of kombucha-like beverage and bacterial cellulose by acerola byproduct as raw material. LWT – Food Sci Technol. 2021;135:110075. 10.1016/j.lwt.2020.110075

[45] Dima SO Panaitescu DM Orban C Ghiurea M Doncea SM Fierascu RC Bacterial nanocellulose from side-streams of kombucha beverages production: Preparation and physical-chemical properties. Polymers. 2017;9(8):374. 10.3390/polym9080374 30971046 PMC6418918

[46] Binda S, Ouwehand AC. Lactic acid bacteria for fermented dairy products. In: Vinderola G, Ouwehand A, Salminen S, von Wright A, editors. Lactic acid bacteria. Boca Raton, FL, USA: CRC Press; 2019. pp. 175–98. 10.1201/9780429057465-12

[47] Meng J Zhang QX Lu RR. Surface layer protein from *Lactobacillus acidophilus* NCFM inhibit intestinal pathogen-induced apoptosis in HT-29 cells. Int J Biol Macromol. 2017;96:766–74. 10.1016/j.ijbiomac.2016.12.085 28057572

[48] Kushargina R Rimbawan R Dewi M Damayanthi E. Metagenomic analysis, safety aspects, and antioxidant potential of kombucha beverage produced from telang flower (*Clitoria ternatea* L.) tea. Food Biosci. 2024;59:104013. 10.1016/j.fbio.2024.104013

[49] Silva LMR Lima JSS Magalhães FEA Campos AR Araújo JIF Batista FLA Graviola fruit bar added acerola by-product extract protects against inflammation and nociception in adult zebrafish (*Danio rerio*). J Med Food. 2020;23(2):173–80. 10.1089/jmf.2019.0078 31502908

[50] Lima TMFG Silva LMR Sousa PHM Magalhães FE Ricardo NMPS Vieira IGP Bioactive jambu extract (*Acmella ciliata*) as source of spilanthol for the development of a functional vegetable gelatin. Food Biosci. 2024;61:104706. 10.1016/j.fbio.2024.104706

[51] Silva FMR Magalhães FEA Batista FLA Silva LMR Ricardo NMPS Sabino LBS Microencapsulation of green tea (*Camellia sinensis*) phenolic extract: Physical-chemical characterization, antimicrobial and toxicological properties. Food Chem Adv. 2023;3:100360. 10.1016/j.focha.2023.100360

[52] Gallina DA Barbosa PPM Ormenese RCSC Garcia AO. Development and characterization of probiotic fermented smoothie beverage. Rev Cienc Agron. 2019;50(3):378–86. 10.5935/1806-6690.20190045

[53] Onodugo NG Agbo EC Ikwumere CM Onwubuya NP Nnadi IM Ezeja EP Nutritional composition and sensory properties of smoothies produced from pineapple (*Ananus comosus*), watermelon (*Citrus lanatus* Thumb) and mango (*Mangifera indica* L). J Fam. Soc Res. 2022;1(2):100–12. 10.66043/jfsr.v1i2.27

[54] Sahin S, Sumnu SG. Size, shape, volume, and related physical attributes. In: Physical properties of foods. Food Science Text Series. New York, NY, USA: Springer; 2006. pp. 1-37. 10.1007/0-387-30808-3_1

[55] Kidoń M Uwineza PA. New smoothie products based on pumpkin, banana, and purple carrot as a source of bioactive compounds. Molecules. 2022;27(10):3049. 10.3390/molecules27103049 35630528 PMC9146844

[56] Sucita T Nilawati UA Latifah R Mardiana A. Prihantono, Andi A. Organoleptic test smoothies Ambon banana fruit (*Musa acuminata*) and cantaloupe (*Cucumis melo* var. *cantaloupe*). IOP Conf Ser Earth Environ Sci. 2023;1230:012049. 10.1088/1755-1315/1230/1/012049

